# Intestinal Absorption of Ergostane and Lanostane Triterpenoids from *Antrodia cinnamomea* Using Caco-2 Cell Monolayer Model

**DOI:** 10.1007/s13659-015-0072-4

**Published:** 2015-09-28

**Authors:** Qi Wang, Xue Qiao, Yi Qian, Zi-wei Li, Yew-min Tzeng, De-min Zhou, De-an Guo, Min Ye

**Affiliations:** State Key Laboratory of Natural and Biomimetic Drugs School of Pharmaceutical Sciences, Peking University, 38 Xueyuan Road, Beijing, 100191 China; Institute of Biochemical Sciences and Technology, Chaoyang University of Technology, Taichung, 41349 Taiwan

**Keywords:** *Antrodia cinnamomea*, Ergostane, Lanostane, Triterpenoids, Caco-2

## Abstract

**Abstract:**

*Antrodia cinnamomea* is a precious medicinal mushroom. It exhibits promising therapeutic effects on cancer, intoxication, hypertension, hepatitis, and inflammation. Its major bioactive constituents are ergostane and lanostane triterpenoids. In this study, we used intestinal Caco-2 cell monolayer model to reveal the intestinal absorption property of 14 representative triterpenoids from *A. cinnamomea*. The bidirectional transport through the monolayer at different time points was monitored by a fully validated LC/MS/MS method. In the case of pure compounds, ergostanes **5** (25*R*-antcin H), **6** (25*S*-antcin H) and **10** (25*R*-antcin B) could readily pass through the Caco-2 cell layer, whereas lanostanes **13** (dehydroeburicoic acid) and **14** (eburicoic acid) could hardly pass through. When the cells were treated with *A. cinnamomea* extract, antcins A, B, C, H and K (**1**–**6** and **9**–**11**) were absorbed via passive transcellular diffusion, and showed high *P*_AB_ and *P*_BA_ values (> 2.5 × 10^−5^ cm/s). Meanwhile, the lanostanes dehydrosulphurenic acid (**8**), 15*α*-acetyldehydrosulphurenic acid (**12**), **13** and **14** exhibited poor permeability. Transport features of these compounds were consistent with their pharmacokinetic behaviors in rats. This study could also be helpful in predicting the intestinal absorption of *A. cinnamomea* in human.

**Graphical Abstract:**

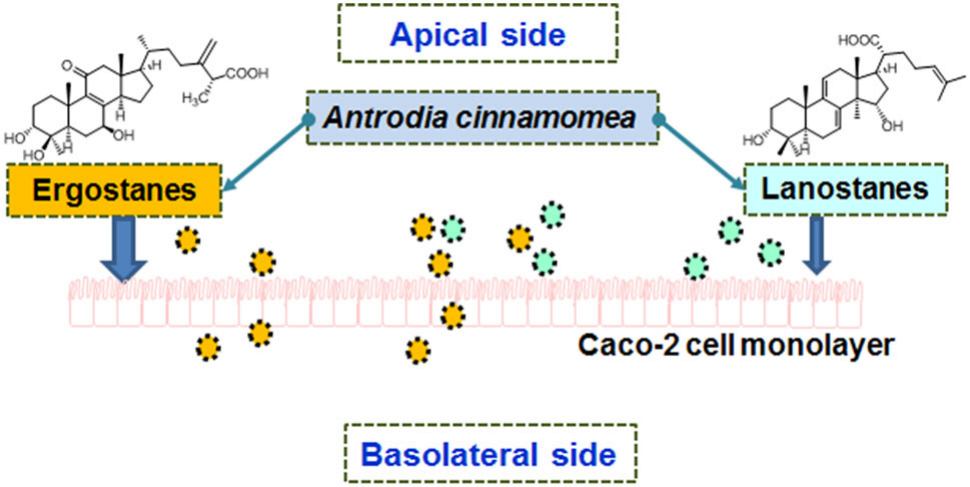

**Electronic supplementary material:**

The online version of this article (doi:10.1007/s13659-015-0072-4) contains supplementary material, which is available to authorized users.

## Introduction

*Antrodia cinnamomea* (or *A. camphorata*, Polyporaceae family, known as “Niu-Chang-Chih” in Chinese) is a precious medicinal mushroom. It has been reported to exhibit anticancer, antioxidant, anti-inflammatory, anti-angiogenesis, liver protection and radioprotection activities [1–5]. Ergostane and lanostane tetracyclic triterpenoids account for 5–15 % of the dry weight, and are the major bioactive constituents of *A. cinnamomea* [6–8]. For instance, antcin K could inhibit the metastasis of human hepatoma cells through suppression of integrin-mediated adhesion, migration, and invasion [9]. Antcin C could protect liver cells from oxidative stress and cell death by increasing HO-1 and Nrf2 expression in mice liver tissue [10].

Recently, we have reported the metabolism and pharmacokinetics of triterpenoids of *A. cinnamomea* in rats [11]. Interestingly, we found that the lanostanes and ergostanes showed remarkably different pharmacokinetic patterns, which were closely related with their chemical structures. High-polarity ergostanes (antcins H (**5**/**6**) and K (**1**/**2**), with two or three hydroxyl groups, respectively) were the major plasma-exposed components. The low-polarity ergostanes antcins B (**9**/**10**) and C (**3**/**4**), containing zero or one hydroxyl group, respectively, could also get into circulation, though exhibiting much lower plasma concentrations. The Δ^7,9(11)^ lanostanes remained in the plasma at a low concentration for a relatively long time. The Δ^8^ lanostanes, however, could not get into circulation. The pharmacokinetic patterns of different types of triterpenoids may be correlated with their intestinal absorption.

The Caco-2 cell monolayer is a widely accepted in vitro model for human intestinal absorption [12, 13]. Therefore, we used the Caco-2 cell monolayer model to interpret the pharmacokinetics of *Antrodia* triterpenoids in rats, and to predict their intestinal absorption in humans. In this work, the bidirectional transport experiments of 14 triterpenoids (**1**–**14**) were studied in *A. cinnamomea* extract, and were compared to five representative pure triterpenoids (**5**, **6**, **10**, **13** and **14**) using the Caco-2 monolayer model. Drug concentrations of apical and basolateral sides (from 30 to 180 min) were determined by a fully validated LC/MS/MS method.

## Results and Discussion

### Method Validation for LC/MS/MS Analysis

**Fig. 1:**
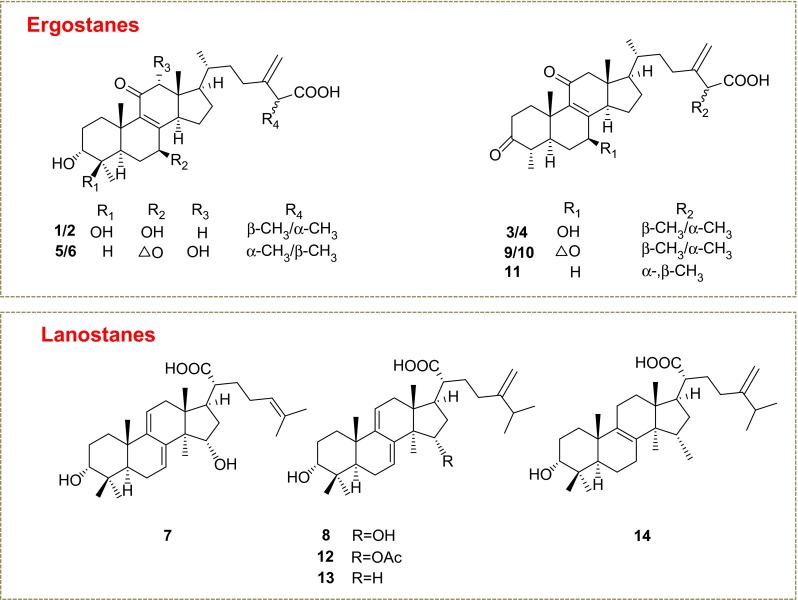
Chemical structures of 14 triterpenoids used in this study. 25*S*-antcin K (**1**), 25*R*-antcin K (**2**), 25*S*-antcin C (**3**), 25*R*-antcin C (**4**), 25*R*-antcin H (**5**), 25*S*-antcin H (**6**), 3*β*,15*α*-dihydroxylanosta-7,9(11),24-triene-21-oic acid (**7**), dehydrosulphurenic acid (**8**), 25*S*-antcin B (**9**), 25*R*-antcin B (**10**), (*R*/*S*)-antcin A (**11**), 15*α*-acetyldehydrosulphurenic acid (**12**), dehydroeburicoic acid (**13**), eburicoic acid (**14**)

**Fig. 2:**
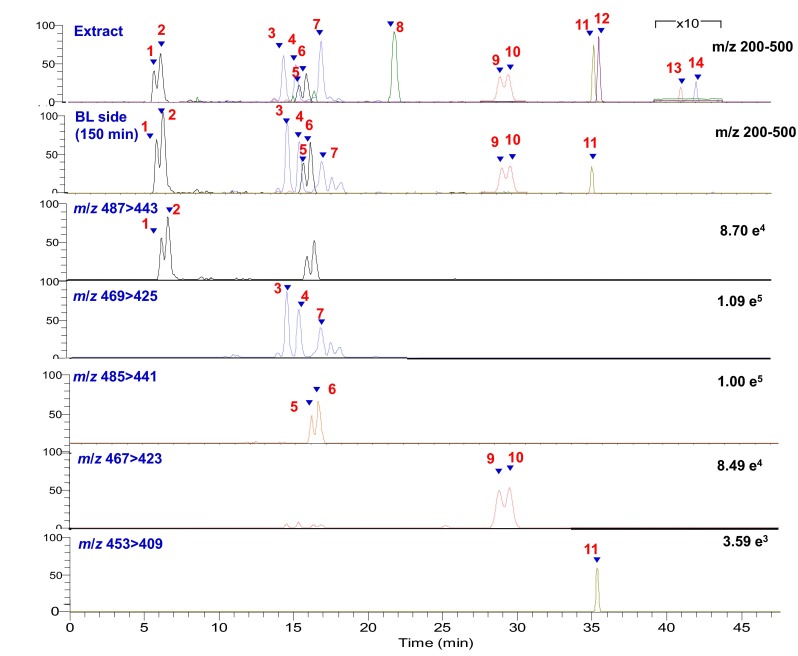
EIC chromatograms of 14 triterpenoids in *A. cinnamomea* extract and in basolateral side at 150 min by LC/MS/MS analysis

To study the bidirectional transport of triterpenoids, the Caco-2 cell monolayers were treated with *A. cinnamomea* extract, or with five pure compounds (**5**, **6**, **10**, **13** and **14**). Concentrations of 14 major triterpenoids (**1**–**14**) (Fig. 1), including nine ergostanes (**1**–**6** and **9**–**11**) and five lanostanes (**7**, **8** and **12**–**14**), in culture medium of the apical and basolateral sides were determined by LC/MS/MS (Fig. 2). The triterpenoids were detected in the selected reaction monitoring (SRM) mode. The HPLC conditions and SRM settings were the same as our pharmacokinetic study [11].

No interfering peaks were observed in the chromatogram of blank HBSS medium samples, indicating good specificity of the detection method. The linearity of each calibration curve was determined by plotting the analyte/internal standard peak area ratio (Y) against the nominal concentration of analytes (X). The calibration curves showed good linearity with correlation coefficients (*r*^2^) of above 0.99. The regression equations and dynamic ranges were listed in Table S1. Accuracy and precision of the method was assessed by testing QC samples at three different concentrations in the same day for five times and on three consecutive days. The intra- and inter-day precisions ranged from 0.87 to 15.66 and 0.56 to 13.41 %, respectively (Table S2). The accuracy was between 86.46 and 114.83 % for LQC, MQC, and HQC, except that the accuracy of **10** in HQC was 83.22 % and **12** in LQC was 84.72 %.

The extraction recoveries and the matrix effects of analytes were determined as we had previously reported [11]. The method recoveries were determined by comparing the measured concentration of un-extracted QC samples (analytes were diluted in methanol and added to freeze-dried HBSS matrix) to the nominal concentration. The extraction and method recoveries at three different QC concentrations were between 85.14–119.73 and 85.73–119.94 % (Table S3). The matrix effects were not significant for most analytes, with ion suppression ranged from −11.94 to 9.01 % (Table S3). Stability was determined at high and low concentrations after 3-day storage at −80 °C. All the analytes showed variations between −18.33 and 10.38 %, indicating the samples were stable within 3 days (Table S4).

### Validation of the Caco-2 Monolayer Model

#### Transepithelial Electrical Resistance (TEER)

Before all permeation experiments, integrity of the Caco-2 membrane was assessed by measuring the TEER values with Millicells^®^ ERS-2 (Millipore, USA) after cell implantation for 0, 2, 4, 8, 12, 14, 16, 18 and 21 days. The TEER values of the Caco-2 cell monolayer should not be lower than 500 Ω·cm^2^. In our experiments, the TEER values reached 532.7 Ω·cm^2^ on day 14, and finally reached 589.3 Ω·cm^2^ on day 21 (Fig. S1).

#### Leakage Marker Fluorescein

The integrity of cell monolayers was also assessed by the transport of the leakage marker sodium fluorescein. On day 21, the Caco-2 monolayers were rinsed twice with pre-warmed HBSS and were then incubated with the same solution at 37 °C for 30 min, on a shaker at 50 rpm. The fluorescein was pre-dissolved in 1 mM NaOH/H_2_O (1:10, *v*/*v*), and then diluted to 1 mM with an appropriate volume of HBSS. The sodium fluorescein and blank HBSS were added to the AP (0.5 mL) and BL side (1.5 mL), respectively. At 30, 60, 90, and 120 min, each 0.3 mL of the solution from BL side was collected, and replaced with an equal volume of HBSS. The sodium fluorescein intensity was determined with a Hitachi F-4500 fluorometer (excitation wavelength 490 nm, emission 525 nm). As shown in Table S5, the *P*_app_ value of sodium fluorescein for AP to BL side was less than 1.0 × 10^−6^ cm/s, indicating that integrity and permeability of the cell monolayer was desirable [14].

#### Alkaline Phosphatase Activity

Alkaline phosphatase activity was determined in the cytosol of Caco-2 cells. The cell monolayer was washed three times with PBS, and then collected into 1 mL homogenizing buffer (0.25 M sucrose in 10 mM Tris–HCl pH 7.4). Cells were centrifuged (5 min, 10000×*g*) and re-suspended in 100 µL of homogenizing buffer before being disrupted by five freeze/thaw cycles in liquid nitrogen and 37 °C water bath. The cytosol was collected after centrifugation (20 min, 10000×*g*, 4 °C) and stored at −80 °C before analysis. The total intracellular protein was determined by the Lowry method, and an AKPase kit was used to determine alkaline phosphatase activity [15]. Alkaline phosphatase activity was calculated as follows:

$${\text{AKP(}}U/L )= (\Delta {\text{A}}/\hbox{min} \, \times V_{\text{t}} \times 1000)/(e \times V_{\text{s}} \times d)$$

ΔA/min represents variety of absorbance per minute, *e* = 8.5 (absorbency per mole), *V*_t_ is the total volume of the mixture (mL), *V*_s_ is volume of the sample (mL), and *d* is thickness of sample cell.

The absorbance of each well was measured at 520 nm by the Infinite^®^ M200 Pro (Tecan, Mannedorf, Switzerland). Alkaline phosphatase activity was calculated using the above formula and expressed as *U*/*g* protein. *U*/*g* was defined as that 1 mg of phenol was generated when 1 g of cellular protein reacted chemically with substrate for 15 min at 37 °C. The results of alkaline phosphatase activity of the Caco-2 cell monolayers were illustrated in Table S6. The activity increased from 309.8 *U*/*g* protein (day 8) to 444.8 *U*/*g* protein on day 15 and 533.4 *U*/*g* protein on day 21, which was consistent with previous reports [15].

### Bidirectional Transport Experiments of Single Triterpenoids

Considering the complex chemical composition of *A. cinnamomea*, the transport features of five single compounds (**5**, **6**, **10**, **13** and **14**) were studied. These five compounds belong to high-polarity ergostanes (**5** and **6**), low-polarity ergostanes (**10**), Δ^7,9(11)^ lanostanes (**13**), and Δ^8^ lanostanes (**14**), respectively, and represent the major types of *A. cinnamomea* compounds. In the Caco-2 cell monolayer model, permeability was described by *P*_AB_ values (the apparent permeability coefficients from AP to BL side). The *P*_AB_ values of well-transported and poorly transported compounds were > 1 × 10^−5^ and < 1 × 10^−6^ cm/s, respectively [16, 17]. *P*_AB_ values of the marker compounds propranolol (3.81 ± 0.34 × 10^−5^ cm/s) and atenolol (7.76 ± 4.65 × 10^−7^ cm/s) in our experiments were consistent with previous reports (Table S5). The *P*_AB_ values of **5**, **6** and **10** (as pure compounds) across Caco-2 cells were all greater than 1 × 10^−5^ cm/s (Table 1), indicating good permeability. Moreover, the efflux ratios (*P*_BA_/*P*_AB_) of **5**, **6**, and **10** were > 2.0 (2.26, 2.05 and 2.07, respectively), indicating an involvement of apical efflux transporters. Their transportation could involve active transport [18–20]. **10** showed lower *P*_AB_ and *P*_BA_ values than **5**/**6**. This might be related to its lower polarity (containing one hydroxyl group), or due to its metabolism. We detected two hydrogenated metabolites of **10** in the AP side (**M1** and **M2**, Fig. S2). The metabolism was consistent with our metabolic studies in rats [11], and could be a factor to change its transport behavior. In contrast to ergostanes, lanostanes **13** and **14** could not be detected in all samples of different time points, indicating poor permeability through the cell monolayer. This could also result from poor water solubility.

**Table 1:** *P*_app_ values, efflux ratio, and log P values of **1**–**7**, and **9**–**11** in *A. cinnamomea* extract, and of **5**, **6**, and **10** as single compounds

Analyte	Concentration (µM)	*P*_app_ (×10^−5^ cm/s)		Efflux ratio	Log P
		*P*_AB_	*P*_BA_	*P*_BA_/*P*_AB_	
In *A. cinnamomea* extract					
**5**	2.61	3.95 ± 0.64	3.47 ± 0.36*^#^	0.88	3.69
**6**	3.66	3.18 ± 0.34	3.05 ± 0.38	0.99	3.69
**10**	2.82	4.11 ± 0.92^#^	3.86 ± 0.32*	0.94	4.37
**1**	1.69	7.80 ± 0.86	5.32 ± 0.31*	0.68	3.07
**2**	1.85	5.74 ± 0.27	4.48 ± 0.30*	0.78	3.07
**3**	1.43	6.82 ± 0.59	4.99 ± 0.29*	0.73	4.04
**4**	0.63	7.41 ± 1.90	5.13 ± 0.27*	0.69	4.04
**7**	0.67	3.74 ± 0.43	3.57 ± 0.38	0.96	5.57
**9**	4.84	2.65 ± 0.53	2.76 ± 0.27	1.04	4.37
**11**	0.82	7.92 ± 0.42	4.79 ± 0.23*	0.6	4.68
As single compounds					
**5**	10	3.72 ± 0.44	8.41 ± 1.35*	2.26	
**6**		3.14 ± 0.56	9.20 ± 1.34*	2.05	
**10**		2.83 ± 0.43	5.85 ± 0.58*	2.07	

25*S*-antcin K (**1**), 25*R*-antcin K (**2**), 25*S*-antcin C (**3**), 25*R*-antcin C (**4**), 25*R*-antcin H (**5**), 25*S*-antcin H (**6**), 3*β*,15*α*-dihydroxylanosta-7,9(11),24-triene-21-oic acid (**7**), 25*S*-antcin B (**9**), 25*R*-antcin B (**10**), 25*R*/*S*-antcin A (**11**)

^#^ *P* < 0.05, extract versus single compound; * *P* < 0.05, *P*_AB_ versus *P*_BA_

### Bidirectional Transport Experiments of *A. cinnamomea* Extract

Based on the study of bidirectional transport of five represent single compounds, the Caco-2 cell monolayer was treated with *A. cinnamomea* extract, and the transportation of 14 triterpenoids (**1**–**14**) were studied.

**Fig. 3:**
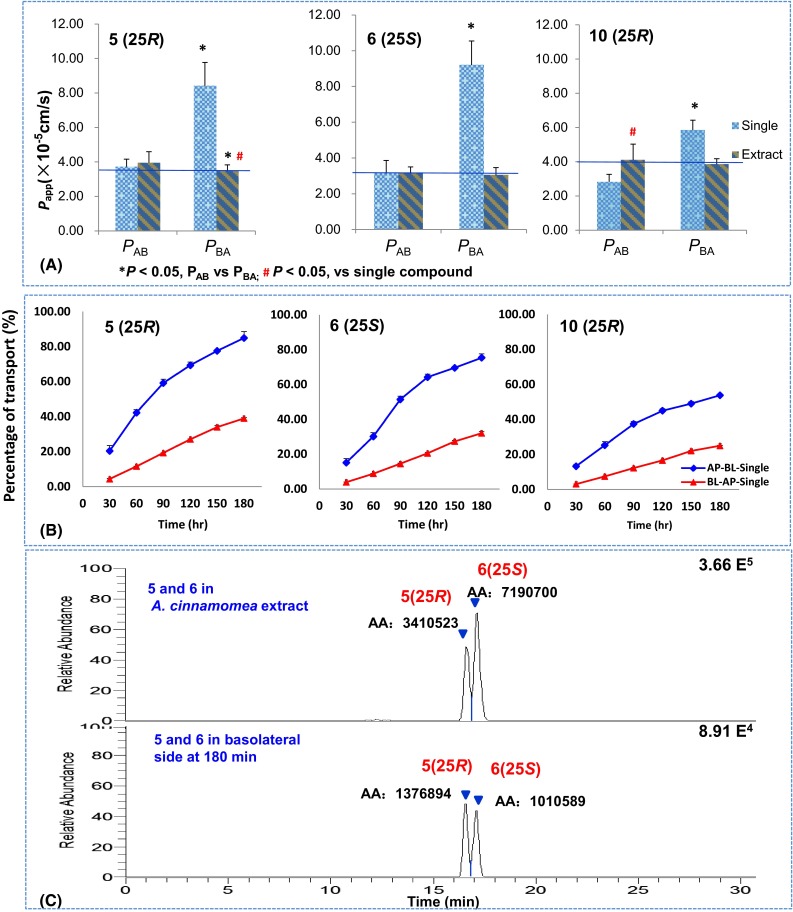
The apparent permeability coefficients (**a**) and transport rates (**b**) of **5**, **6** and **10**, and LC/MS/MS chromatograms of **5**/**6** in *A. cinnamomea* extract and in basolateral side (**c**)

Compared to the single form, *P*_BA_ values of **5**, **6**, and **10** in *A. cinnamomea* extract (containing 2.61, 3.66, 2.82 µM of **5**, **6**, and **10**, respectively) decreased significantly by 50–60 % to 3.0–3.8 × 10^−5^ cm/s (Fig. 3a). However, no significant difference was observed for the *P*_AB_ values. Accordingly, efflux ratios of **5**, **6**, and **10** decreased to lower than 1.0 (0.88, 0.99, and 0.94, respectively), indicating their intestinal absorptions were dominated by passive diffusion [20, 21] (Table 1). These data suggested that *A. cinnamomea* might contain transporter inhibitors (e.g. P-gp or MRP inhibitors) [19]. Moreover, though 25*R*/*S*-antcin H (**5**/**6**) were epimers, they showed different transport capacities. The transport rate of **5** was higher than that of **6**, both in single form and in the extract (Fig. 3b). As a result, although the content of **5** was lower than **6** in *A. cinnamomea* extract, its concentration was higher than **6** in the basolateral side (Fig. 3c).

**Fig. 4:**
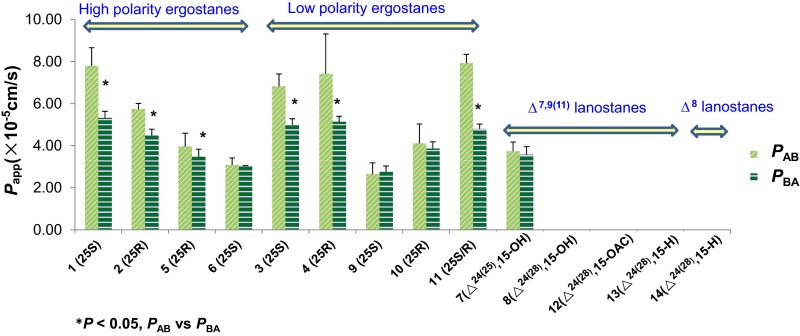
The apparent permeability coefficients of **1**–**14** in *A. cinnamomea* extract

**Fig. 5:**
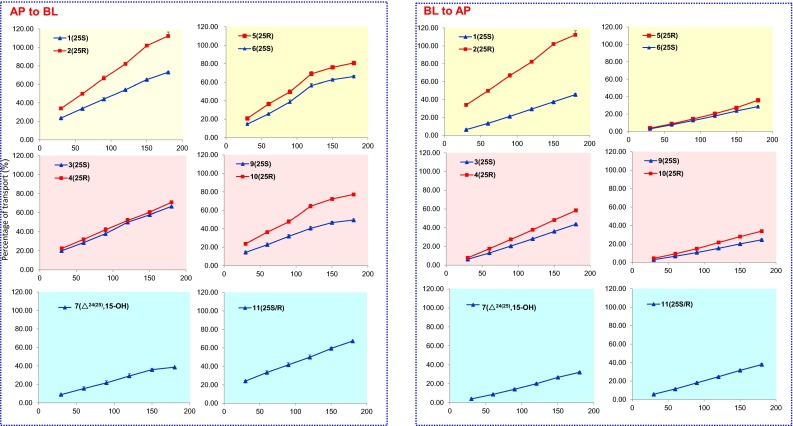
Time-transport rate curves of **1**–**7** and **9**–**11** in *A. cinnamomea* extract

The *P*_app_ values of ergostanes **1**–**4**, **7**, **9** and **11** were summarized in Table 1 and Fig. 4. They showed favorable permeability (*P*_app_ > 2.5 × 10^−5^ cm/s). Ergostanes have high polarity than lanostanes, and their log P values were between 3 and 4.7. This was consistent with previous reports [12, 22], where the compounds with moderate log P values (3–4) exhibited higher permeability. Meanwhile, among different ergostanes, the permeability was not significantly correlated with log P values, though the polarity (number of hydroxyl groups) was inversely correlated with log P values. To all these ergostanes, their efflux ratio (≤1.0) indicated that they were absorbed mainly by passive diffusion [20, 21]. Moreover, as shown in Fig. 5, we found the transport rates of 25*R* ergostane epimers (**2**, **4**, **5** and **10**) from AP to BL side were significantly higher than 25*S* ergostane epimers (**1**, **3**, **6** and **9**) in *A. cinnamomea* extract (Fig. 5). Since that the absorption rates are closely related to physico-chemical properties (such as log P and hydrogen bonding capacity) of the drug molecule [12], the chemical environment of C-25 might be an important factor for transportation. In addition, the transport rate of **2** was higher than 100 % (112 %), probably due to the transformation of other compounds into **2** [11].

Δ^7,9(11)^ lanostanes (**8**, **12**, **13**) and Δ^8^ lanostane (**14**) exhibited very low solubility and permeability, both in the extract and single forms (**13** and **14**). Log P values of **8**, **12**, **13** and **14** were 5.45, 5.44, 6.17 and 6.09, respectively. This was consistent with previous reports, where compounds with higher log P values (> 4.5) showed significantly lower permeability than the ones with moderate log P values (3–4) [12, 20]. Nonetheless, compound **7** could pass through the Caco-2 cell monolayer with *P*_AB_ value of 3.74 ± 0.43 × 10^−5^ cm/s, though its transport rate (38.8 % at 180 min) were lower than ergostane triterpenoids (> 49.5 % at 180 min).

## Conclusion

In this work, absorption properties of 14 ergostane and lanostane triterpenoids in *A. cinnamomea* were investigated using the Caco-2 cell monolayer model. The concentrations were determined by using a fully validated LC/MS/MS method. As pure compounds, ergostanes 25*R/S*-antcin H (**5**/**6**) and 25*R*-antcin B (**10**) exhibited high permeability, and their transportation might involve efflux transporters (efflux ratio > 2.0). When the cell monolayer was treated with *A. cinnamomea* extract, all ergostanes (antcins A, B, C, H, K, log P 3.0–4.7) showed favorable permeability (*P*_app_ > 2.5 × 10^−5^ cm/s), and they were mainly absorbed via passive diffusion (efflux ratio ≤ 1.0). Transportation rate of 25*R*-ergostanes was higher than 25*S*-ergostanes. Meanwhile, lanostanes (log P > 5.4) were barely permeable. The transport features of ergostane and lanostane triterpenoids through the Caco-2 cell monolayer were consistent with their pharmacokinetic behaviors. This study provided information in predicting the intestinal absorption of *A. cinnamomea* in human.

## General Experimental Procedures

### Chemicals and Reagents

The fruiting bodies of *A. cinnamomea* were cultivated by Professor Yew-Min Tzeng at Chaoyang University of Technology, Taiwan. A voucher specimen was deposited at School of Pharmaceutical Sciences, Peking University, Beijing, China. Reference compounds **1**–**14** were isolated by the authors [6]. The purities were above 98 % as determined by HPLC/UV analysis. Their structures are given in Fig. 1. HPLC-grade acetonitrile, methanol and formic acid were purchased from Mallinckrodt Baker (Phillipsburg, NJ, USA). Ultra-pure water was prepared with a Milli-Q water purification system (Millipore, Billerica, MA, USA). The other reagents were of analytical grade.

Fetal bovine serum (FBS) was purchased from PAA Laboratories GmbH (Linz, Austria). Penicillin and streptomycin solutions (10000 *U*/mL penicillin and 10000 mg/mL streptomycin) and Hank’s Balanced Salts Solution (HBSS) were obtained from M&C Gene Technology Co., Ltd. (Beijing, China). Non-essential amino acids, l-Glutamine and Trypsin–EDTA (0.25 % (*w*/*w*) trypsin/1 mM EDTA) were all from Gibco Laboratories (Life Technologies Inc., USA). Ethylene diamine tetraacetic acid (EDTA), dimethyl sulfoxide (DMSO), fluorescein, propranolol, and atenolol with purity of minimum 98 % were products of Sigma-Aldrich (St. Louis, MO, USA). AKPase kit (JC-A0059) was purchased from Nanjing Jiancheng Bioengineering Research Institute (Nanjing, Jiangsu, China). Transwell™ plates of 12 wells (12 mm membrane diameter, 3.0 μm pore size, 1.12 cm^2^ surface area) and 96-wells plates were obtained from Corning Costar (Cambridge, MA, USA).

### Sample Preparation

Representative compounds **5**, **6**, **10**, **13** and **14** were respectively dissolved in DMSO (9 µL) and then diluted with HBSS (3 mL) to the concentration at 10 µM as cell treatment solution. The final concentrations of DMSO were controlled below 0.3 % (*v*/*v*) to ensure safety to the cells [23].

The dried fruiting bodies of *A. cinnamomea* (30 g) were powdered and extracted with 500 mL ethanol for three times (2 h for each time) under reflux. The extracts were combined, concentrated *in vacuo*, dispersed in H_2_O and freeze-dried to obtain the *A. cinnamomea* extract (33.3 % yield). Before the bidirectional transport experiments, 250.13 µg *A. cinnamomea* extract was suspended in 0.03 mL DMSO and then diluted with HBSS to prepare the extract solution (25 µg/mL).

### Cell Culture

The human colon adenocarcinoma cell line Caco-2 was purchased from the Cell Resource Center, Peking Union Medical College (CRC/PUMC, China), and was cultured in a humidified atmosphere of 5 % CO_2_ at 37 °C. Caco-2 cells were cultured in DMEM with 10 % FBS (inactivation at 56 °C for 30 min), 1 % NEAA, and 1 % penicillin and streptomycin solution. The culture medium was changed every other day during cell growth and differentiation. On achieving 80–90 % confluence, the cells were rinsed with 4 mL pre-warm PBS (pH 7.4) and split using 1 mL trypsin. The cells were then seeded on polycarbonate membranes of cell culture inserts at a density of 1.5 × 10^5^ cells/mL and placed in 12 well cell culture clusters. The transepithelial electrical resistance (TEER) using a Millicells^®^ ERS-2 (Millipore, USA) and accumulation of fluorescein were assessed to reflect the tightness of intercellular junctions [24]. Reference compounds propranolol and atenolol were used to validate the Caco-2 cell monolayer model. They represent paracellular flux and transcellular flux compounds, respectively [16, 17]. Differentiation of Caco-2 cells was checked on days 8, 15, 21 by determining the alkaline phosphatase activity using the AKP assay kit, a brush border enzyme marker [15, 25]. The Caco-2 cell monolayers were used for transport experiments on day 21 post-seeding with TEER values >500 Ω·cm^2^.

### Bidirectional Transport Experiments

Caco-2 cells were obtained from CRC/PUMC at passage 18 and all experiments were performed from passages 24–35. Before the transport experiments, the cell monolayer was washed three times with HBSS. Then, the plates were incubated in fresh HBSS for 30 min at 37 °C. The experiments were conducted by adding the samples (pure compound solution (10 µM) and *A. cinnamomea* extract solution (25 µg/mL), respectively) to either the apical (AP, 0.5 mL) or basolateral side (BL, 1.5 mL), while the receiving chamber contained the corresponding volume of pre-warmed HBSS. Every experiment was repeated three times, and the plates were incubated in an orbital shaker at 37 °C, 50 r/min. To assess the drug transport, at the incubation time of 30, 60, 90, 120, 150 and 180 min, a 0.3 mL aliquot was removed and was immediately replenished with an equal volume of HBSS [15, 18]. The samples were freeze-dried and then dissolved in 0.3 mL of methanol. The solution was filtered through a 0.22-µm membrane for LC/MS/MS analysis. The transport rate was calculated as the ratio of cumulative concentration in the receiver to the donor side × 100 %. The apparent permeability coefficient was indicated by the absorption rate constant *P*_app_. It could be calculated as *P*_BA_ (measured in BL to AP direction) or *P*_AB_ (measured in AP to BL direction), using the equation of *P*_app_ = (d*Q*/d*t*)/(A × C_0_). d*Q*/d*t* is the rate at which the compound appears in the receiver chamber (μmol/s), A is the surface area of the filter membrane (1.12 cm^2^) and C_0_ is the initial concentration in the donor chamber (μmol/mL). Efflux ratio (ER) was calculated by the equation of ER = *P*_BA_/*P*_AB_, where *P*_BA_ is the *P*_app_ value measured in BL to AP direction, and *P*_AB_ is the *P*_app_ value measured in AP to BL direction.

When *A. cinnamomea* extract was added into the Caco-2 cultures at 25 µg/mL, the final concentrations of compounds **1**–**14** were 1.69, 1.85, 1.43, 0.63, 2.61, 3.66, 0.67, 1.04, 4.84, 2.82, 0.82, 0.80, 0.46, and 0.22 µM, respectively, as determined by LC/MS/MS analysis (Fig. 2). The pure compounds **5**, **6**, **10**, **13** and **14** were added into the Caco-2 cultures at a final concentration of 10 µM. MTS assay indicated that *A. cinnamomea* extract and the pure compounds were non-toxic to the cells at 25 µg/mL and 10 µM, respectively (data not shown).

### Calibration Standards and Quality Control Solutions

Reference standard compounds were dissolved in DMSO to prepare individual stock solutions (70 µM for **1**–**4**, **7** and **9**; 140 µM for **8**, **10**, **11**, **13** and **14**; 350 µM for **5**, **6** and **12**). These stock solutions were mixed and then serially diluted using HBSS to obtain calibration standard solutions (20, 10, 5, 2.5, 1, 0.5, 0.2, and 0.1 µM for each compound). Quality control (QC) stock solutions were prepared at three concentration levels as high QC (HQC), middle QC (MQC), and low QC (LQC), based on linear ranges of the analytes. All solutions were sealed and stored at −20 °C until use.

### LC/MS/MS Analysis

The LC/MS/MS method we had previously established for the pharmacokinetics study of *A. cinnamomea* was used [11]. Data were processed by Xcalibur 2.0.7 software (ThermoFisher).

### Statistical Analysis

Results were expressed as the mean ± SD, of which the mean value was the average of at least three replicates. Analysis of variance (ANOVA) was used to test the statistical significance of differences between groups. Statistical significance in the differences of the means was determined by Student’s *t* test, with a significance level of *P* < 0.05. The logarithm of octanol–water partition coefficient (log P) was calculated with Pallas 3.3 (CompuDrug International, Inc., USA).

## Electronic supplementary material

Supplementary material 1 (PDF 485 kb)
