# Supplementary material for: Intestinal Absorption of Ergostane and Lanostane Triterpenoids from Antrodia cinnamomea Using Caco-2 Cell Monolayer Model
Source: Nat Prod Bioprospect. 2015 Sep 28;5(5):237–46. doi: 10.1007/s13659-015-0072-4 (PMC4607679; doi:10.1007/s13659-015-0072-4)
Supplement: Supplementary file 1 — Supplementary material 1 (PDF 485 kb) [file 13659_2015_72_MOESM1_ESM.pdf]

Supporting Information

Intestinal absorption of ergostane and lanostane triterpenoids from *Antrodia cinnamomea* using Caco-2 cell monolayer model

Qi Wang[1,Δ], Xue Qiao[1,Δ], Yi Qian[1], Zi-wei Li[1], Yew-min Tzeng[2], De-min Zhou[1,*], De-an Guo[1], Min Ye[1,*]

[1] *State Key Laboratory of Natural and Biomimetic Drugs, School of Pharmaceutical Sciences, Peking University, Beijing 100191, China.*

[2] *Institute of Biochemical Sciences and Technology, Chaoyang University of Technology, Taichung 41349, Taiwan.*

[*] Corresponding author: State Key Laboratory of Natural and Biomimetic Drugs, School of Pharmaceutical Sciences, Peking University, 38 Xueyuan Road, Beijing 100191, China. Tel.: +86 10 82801516; Fax: +86 10 82802024; E-mail address: yemin@bjmu.edu.cn (M. Ye), deminzhou@bjmu.edu.cn (D.M. Zhou).

Table of Contents

Table S1. Calibration curves for Caco-2 cell monolayer model.

Table S2. Intraday and interday variations of 14 triterpenoid analytes (*n* = 5).

Table S3. Matrix effect, extraction and method recoveries of the analytes (*n* = 3).

Table S4. Stabilities of the analytes (*n* = 3).

Table S5. The P_{app} values from AP to BL side of sodium fluorescein, propranolol and atenolol in the Caco-2 cell monolayer model.

Table S6. Alkaline phosphatase activity of the developing Caco-2 monolayer as the function of time.

Fig. S1. TEER values of the developing Caco-2 monolayer as the function of time.

Fig. S2. EIC chromatograms of compound **10** and its metabolites in the apical side.

Table S1. Calibration curves for Caco-2 cell monolayer model.

Analyte	Calibration equation	Range (nM)	r^2
**1**	Y = 1110.7X - 46974	100-5000	0.9998
**2**	Y = 1835.8X + 69520	100-5000	0.9991
**3**	Y = 1983X - 55175	100-5000	0.9990
**4**	Y = 3520.2X - 53635	100-5000	0.9995
**5**	Y = 1687X - 320137	500-20000	0.9990
**6**	Y = 1845.8X - 154136	500-20000	0.9987
**7**	Y = 27795X - 1E+06	100-5000	0.9994
**8**	Y = 109.81X - 34565	200-10000	0.9988
**9**	Y = 5282.2X - 273481	100-5000	1.0000
**10**	Y = 6134.8X - 1E+06	200-10000	0.9992
**11**	Y = 2397.9X - 185413	200-10000	0.9990
**12**	Y = 157.63X - 52728	500-20000	0.9994
**13**	Y = 612.38X+ 115657	200-10000	0.9997
**14**	Y = 612.61X - 38670	200-10000	0.9994

Table S2. Intraday and interday variations of 14 triterpenoid analytes (*n* = 5).

		Analyte													
		**1**	**2**	**3**	**4**	**7**	**9**	**8**	**10**	**11**	**13**	**14**	**5**	**6**	**12**
HQC(nM)															
	NC	3500.00	3500.00	3500.00	3500.00	3500.00	3500.00	7000.00	7000.00	7000.00	7000.00	7000.00	17500.00	17500.00	17500.00
Intraday	MC	3840.90	3935.66	3428.99	3312.18	3515.99	3798.04	7642.68	6552.44	7366.27	7649.00	7994.02	17927.88	17596.75	15129.92
	RSD	1.65	1.81	6.52	2.35	2.48	2.36	5.01	5.36	5.54	5.95	7.92	2.22	2.81	6.32
	A	109.74	112.45	97.97	94.63	100.46	108.52	109.18	93.61	105.23	109.27	114.20	102.45	100.55	86.46
Interday	MC	3953.39	4001.92	3344.93	3305.99	3390.63	3978.78	7858.14	6411.09	6450.58	7986.01	7331.50	18349.66	17879.46	15972.25
	RSD	12.07	9.12	9.24	4.69	3.30	8.33	5.25	7.52	11.78	4.63	2.45	8.26	7.48	7.45
	A	112.95	114.34	95.57	94.46	96.88	113.68	112.26	83.22	92.15	114.09	104.74	104.86	102.17	91.27
Accuracy	RE	9.50	14.34	-4.55	-4.87	-1.23	9.61	12.26	-8.41	13.02	14.09	4.74	2.15	0.22	-8.73
	RSD	7.17	1.91	1.72	1.73	2.70	2.21	5.28	5.03	3.07	6.98	8.27	2.45	3.07	5.77
MQC(nM)															
	NC	1050.00	1050.00	1050.00	1050.00	1050.00	1050.00	2100.00	2100.00	2100.00	2100.00	2100.00	5250.00	5250.00	5250.00
Intraday	MC	1140.53	1177.57	1150.73	1205.68	1201.06	1160.56	2252.76	1942.46	2391.85	2371.76	2402.36	5989.41	6002.97	5185.86
	RSD	3.12	4.35	5.71	3.80	2.45	3.35	9.42	0.87	5.48	15.66	13.08	2.35	1.50	7.43
	A	108.62	112.15	109.59	114.83	114.39	110.53	107.27	92.50	113.90	112.94	114.40	114.08	114.34	98.78
Interday	MC	1201.51	1125.31	1080.36	1135.91	1161.04	1171.25	2313.69	1848.81	2312.26	2184.44	2389.55	5986.32	5953.63	4964.40
	RSD	2.98	0.56	10.21	7.45	6.51	5.13	11.64	1.69	1.75	7.33	3.74	2.12	1.81	2.16
	A	114.43	107.17	102.89	108.18	110.58	111.55	110.18	88.04	110.11	104.02	113.79	114.03	113.40	94.56
Accuracy	RE	14.43	7.17	7.18	8.41	12.28	12.45	17.91	10.18	10.11	-9.60	11.31	14.03	14.99	-6.24
	RSD	9.93	4.49	2.47	3.70	2.22	7.69	6.89	1.72	8.17	5.52	3.60	2.03	2.95	3.40
LQC(nM)															
	NC	350.00	350.00	350.00	350.00	350.00	350.00	700.00	700.00	700.00	700.00	700.00	1750.00	1750.00	1750.00
Intraday	MC	369.15	354.03	377.33	372.60	401.09	396.36	669.14	679.03	643.52	626.16	802.30	2002.55	1976.04	1629.59

	RSD	8.03	9.23	8.29	6.09	3.09	6.51	6.19	2.80	4.51	4.78	6.86	2.85	1.58	3.79
	A	105.47	101.15	107.81	106.46	114.60	113.25	95.59	97.00	91.93	89.45	114.61	114.43	112.92	93.12
	MC	383.65	331.14	362.63	363.57	395.03	386.89	724.60	695.04	613.93	803.36	799.66	1994.50	1996.71	1482.51
Interday	RSD	7.05	2.77	3.99	11.41	3.50	5.40	1.58	3.56	10.09	9.90	13.41	6.75	1.74	11.51
	A	109.61	94.61	103.61	103.88	112.87	110.54	103.51	99.29	87.70	114.77	114.24	113.97	114.10	84.72
Accuracy	RE	12.08	2.22	7.23	10.18	13.24	13.63	-3.19	-3.20	-12.30	14.77	15.83	13.97	13.73	-8.94
	RSD	6.88	6.00	2.98	3.67	1.80	4.40	3.66	1.32	4.80	14.70	10.83	3.56	2.19	2.65

NC, nominal concentration; MC, measured concentration; A, accuracy in %; RE, relative error in %.

Table S3. Matrix effect, extraction and method recoveries of the analytes (*n* = 3).

		**Analyte**													
		**1**	**2**	**3**	**4**	**7**	**9**	**8**	**10**	**11**	**13**	**14**	**5**	**6**	**12**
HQC(nM)															
	NC	3500.00	3500.00	3500.00	3500.00	3500.00	3500.00	7000.00	7000.00	7000.00	7000.00	7000.00	17500.00	17500.00	17500.00
ER(%)	AVE	96.70	96.53	102.31	101.24	101.83	98.62	96.55	113.16	119.73	88.43	90.79	99.07	99.73	114.84
	RSD	2.42	3.94	5.58	4.53	2.51	6.91	11.43	4.23	7.77	12.87	9.50	1.95	1.58	4.87
MR(%)	AVE	109.50	119.41	95.45	95.13	98.77	109.61	108.13	88.37	113.02	98.38	115.90	102.15	100.22	85.73
	RSD	7.17	1.91	1.72	1.73	2.70	2.21	5.28	6.54	3.07	6.98	8.27	2.45	3.07	5.34
ME(%)	AVE	103.34	98.53	99.36	100.48	99.97	97.70	99.33	105.53	110.44	99.20	101.05	100.40	99.84	106.02
	RSD	2.73	1.04	0.29	0.44	3.31	0.59	2.34	1.89	3.20	3.84	0.58	0.72	0.15	4.71
MQC(nM)															
	NC	1050.00	1050.00	1050.00	1050.00	1050.00	1050.00	2100.00	2100.00	2100.00	2100.00	2100.00	5250.00	5250.00	5250.00
ER(%)	AVE	93.90	89.88	107.65	112.02	106.57	106.61	104.57	115.21	115.09	96.09	91.26	101.60	104.27	114.10
	RSD	13.84	5.67	6.37	13.03	4.95	1.99	11.00	1.41	7.47	10.09	9.86	3.11	4.05	4.09
MR(%)	AVE	115.49	113.64	107.18	108.41	112.28	112.45	117.91	86.30	114.38	90.40	111.31	118.50	114.99	93.76
	RSD	9.93	4.49	2.47	3.70	2.22	7.69	6.89	1.72	8.17	5.52	3.60	2.03	2.95	3.40
ME(%)	AVE	98.71	95.78	103.03	101.48	101.79	105.61	100.12	111.94	99.30	98.98	99.42	100.95	101.61	107.51
	RSD	2.81	3.10	2.37	1.93	1.88	2.17	2.26	0.25	7.74	0.58	2.33	0.47	6.19	1.95
LQC(nM)															
	NC	350.00	350.00	350.00	350.00	350.00	350.00	700.00	700.00	700.00	700.00	700.00	1750.00	1750.00	1750.00
ER(%)	AVE	109.21	108.41	102.83	103.20	99.11	103.69	85.14	101.90	99.60	100.06	85.78	98.50	102.26	95.16
	RSD	11.96	9.40	4.23	4.25	5.58	5.59	6.17	2.01	2.97	3.06	7.62	7.94	5.48	1.56
MR(%)	AVE	112.08	102.22	107.23	110.18	113.24	113.63	96.81	96.80	91.31	116.51	115.83	119.94	113.73	91.06
	RSD	6.88	6.00	2.98	3.67	1.80	4.40	3.66	1.32	4.80	14.70	10.83	3.56	2.19	2.65

ME(%)	AVE	102.05	102.59	99.95	99.79	101.80	98.80	90.99	100.22	99.15	99.71	94.32	99.46	99.26	97.96
	RSD	6.45	4.82	3.17	3.75	4.77	1.92	3.08	0.16	1.20	4.30	3.86	1.48	3.24	0.28

NC, nominal concentration; MC, measured concentration; ER, extraction recovery; MR, method recovery; ME, matrix effects.

Table S4. Stabilities of the analytes (*n* = 3).

	**Analyte**													
	**1**	**2**	**3**	**4**	**7**	**9**	**8**	**10**	**11**	**13**	**14**	**5**	**6**	**12**
HC(nM)														
NC	3500.00	3500.00	3500.00	3500.00	3500.00	3500.00	7000.00	7000.00	7000.00	7000.00	7000.00	17500.00	17500.00	17500.00
0 h MC	3832.51	4179.18	3340.88	3329.47	3456.93	3836.23	7569.07	5364.02	7911.74	6886.47	8112.70	17876.45	17537.71	13214.89
3 d MC	4032.74	4303.98	3368.02	3352.75	3566.86	3874.86	7948.67	4807.04	7396.08	7984.94	9548.21	18280.51	17906.86	12546.79
3 d V	-5.22	-2.99	-0.81	-0.70	-3.18	-1.01	-5.02	10.38	6.52	-15.95	-17.69	-2.26	-2.10	5.06
RSD	3.65	3.63	1.85	3.08	3.38	6.82	6.21	3.34	3.53	7.06	1.84	3.75	3.83	8.11
LC(nM)														
NC	350.00	350.00	350.00	350.00	350.00	350.00	700.00	700.00	700.00	700.00	700.00	1750.00	1750.00	1750.00
0 h MC	392.27	357.78	375.31	385.63	396.35	397.70	677.70	677.63	639.19	815.54	810.83	2098.94	1990.24	1674.74
3 d MC	463.20	408.61	395.25	393.60	417.51	434.90	790.74	694.04	756.34	741.19	914.58	2354.95	2153.24	1937.28
3 d V	-18.08	-14.21	-5.31	-2.07	-5.34	-9.35	-16.68	-2.42	-18.33	9.12	-12.80	-12.20	-8.19	-15.68
RSD	7.89	3.02	5.85	4.11	1.07	3.95	6.23	2.47	6.39	15.67	3.82	2.52	1.92	7.84

NC, nominal concentration; MC, measured concentration; V, variation in %; RSD, relative standard deviation in %; 3 d, 3-day storage.

Table S5. The P_{app} values from AP to BL side of sodium fluorescein, propranolol and atenolol in the Caco-2 cell monolayer model.

Analyst	Time (min)	P_{app} (×10^{-7} cm/s)	Analyst	P_{app} (×10^{-5} cm/s)	Analyst	P_{app} (×10^{-7} cm/s)
Fluorescein	30	7.98 ±1.99	Propranolol	3.51 ±1.06	Atenolol	7.10 ±1.40
	60	7.71 ±1.44		3.76 ±0.45		7.36 ±1.52
	90	7.65 ±1.40		3.43 ±0.46		7.96 ±1.04
	120	7.08 ±2.98		3.81 ±0.34		7.76 ±4.65

Table S6. Alkaline phosphatase activity of the developing Caco-2 monolayer as the function of time

Day	⊿A/min-⊿A_{KB}/min	AKP (*U/L*)	Average (*U/L*)	SD	Protein (μg/mL)	Average (μg/mL)	SD	*U/g* protein
8	0.0618	170.29	151.01	16.76	489.00	487.44	11.37	309.80
	0.0455	125.44			490.00			
	0.0555	153.11			482.00			
	0.0579	159.63			469.00			
	0.0532	146.58			503.33			
	0.0495	136.35			491.33			
15	0.0809	223.01	229.77	13.21	532.20	516.52	18.39	444.84
	0.0771	212.43			523.20			
	0.0897	247.17			531.80			
	0.0832	229.49			525.00			
	0.0859	236.74			489.40			
	0.0822	226.65			497.54			
21	0.1312	361.85	367.67	14.92	685.45	689.24	11.60	533.45
	0.1399	385.67			678.62			
	0.1300	358.49			705.93			
	0.1274	351.28			690.00			
	0.1382	381.08			676.29			
	0.1391	383.60			699.16			

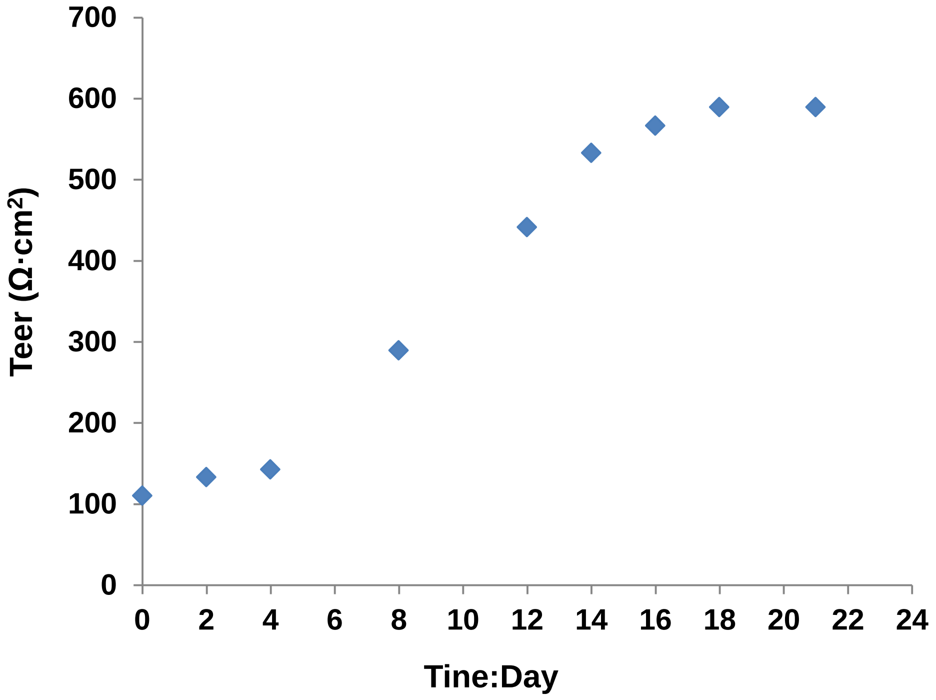

Fig. S1. TEER values of the developing Caco-2 monolayer as the function of time.

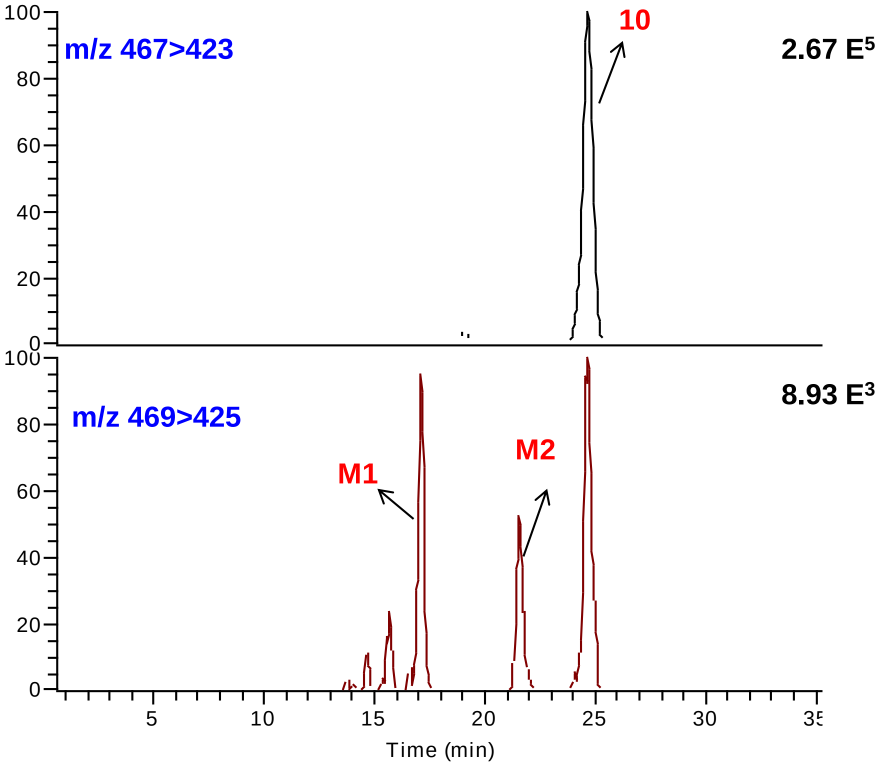

Fig. S2. EIC chromatograms of compound **10** and its metabolites in the apical side.

The sample was collected 120 min following the treatment of single compound (10 μM of **10**).
